# Changes of myocardial lipidomics profiling in a rat model of diabetic cardiomyopathy using UPLC/Q-TOF/MS analysis

**DOI:** 10.1186/s13098-017-0249-6

**Published:** 2017-07-20

**Authors:** Shifen Dong, Rong Zhang, Yaoyue Liang, Jiachen Shi, Jiajia Li, Fei Shang, Xuezhou Mao, Jianning Sun

**Affiliations:** 1 Department of Pharmacology, School of Chinese Materia Medica, Beijing University of Chinese Medicine, No. 6 Wang Jing Zhong Huan South Road, Chaoyang District, Beijing, 100102 China; 2 Beijing University of Chemical Technology, Beijing, 100029 China; 3 Biostatistics and Programming, Sanofi U.S., Bridgewater, NJ 08807 USA

**Keywords:** Diabetic cardiomyopathy, Lipidomics profiling, Myocardial tissue, UPLC/Q-TOF/MS analysis

## Abstract

**Background:**

Diabetic cardiomyopathy (DCM) is a serious cardiac dysfunction induced by changes in the structure and contractility of the myocardium that are initiated in part by alterations in energy substrates. The underlying mechanisms of DCM are still under controversial. The observation of lipids, especially lipidomics profiling, can provide an insight into the know the biomarkers of DCM. The aim of our research was to detect changes of myocardial lipidomics profiling in a rat model of diabetic cardiomyopathy.

**Methods:**

Diabetic cardiomyopathy was induced by feeding a high-sucrose/fat diet (HSFD) for 28 weeks and streptozotocin (30 mg/kg, intraperitoneally). The ultra-high-performance liquid chromatography (UPLC) coupled to quadruple time-of flight (QTOF) mass spectrometer was used to acquire and analyze the lipidomics profiling of myocardial tissue. Meanwhile, parameters of cardiac function were collected using cardiac catheterization, and the cardiac index was calculated, and fasting blood glucose and lipid levels were measured by an ultraviolet spectrophotometric method.

**Results:**

We detected 3023 positive ion peaks and 300 negative ion peaks. Levels of phosphatidylcholine (PC) (22:6/18:2), PC (22:6/18:1), PC (20:4/16:1), PC (16:1/18:3), phosphatidylethanolamine (PE) (20:4/18:2), and PE (20:4/16:0) were down-regulated, and PC (20:2/18:2), PC (18:0/16:0), and PC (20:4/18:0) were up-regulated in DCM model rats, when compared with control rats. Cardiac functions signed as values of left ventricular systolic pressure, maximal uprising velocity of left ventricular pressure and maximal decreasing velocity of left ventricular pressure were injured by 21–44%, and the cardiac index was increased by 25%, and fasting blood glucose and lipids were increased by 34–368%. Meanwhile, the cardiac lipid-related biomarkers have significant correlation with changes of cardiac function and cardiac index.

**Conclusions:**

UPLC/Q-TOF/MS analysis data suggested changes of some potential lipid biomarkers in the development of cardiac dysfunction and hypertrophy of diabetic cardiomyopathy, which may serve as potential important targets for clinical diagnosis and therapeutic intervention of DCM in the future.

## Background

Diabetes mellitus (DM) is a metabolic disorder with increasing prevalence that has serious economic consequences to health care systems [1]. The prevalence of DM is predicted to rise from 175 million in 2000 to 353 million by 2030 [2], in addition, it is estimated that 470 million people will have prediabetes by 2030 [3] Particularly, type 2 diabetes mellitus (T2D) is epidemic worldwide due to the rising rates of obesity worldwide, over one billion people are overweight or obese [4]. DM complications are associated with both microvascular (retinopathy, nephropathy, neuropathy) and macrovascular (atherosclerosis, cardiomyopathy) pathologies [3, 5, 6]. Among of them, cardiovascular diseases represent the primary cause of death in diabetic patients, due to coronary artery disease or associated hypertension [7], but also related to a direct deleterious effect on the myocardium of hyperglycemia, so called diabetic cardiomyopathy (DCM) [8]. The prevalence of diabetic cardiomyopathy is about 12% and reaches 22% in people over 64 years old [9].

Diabetic cardiomyopathy is described as the structural and functional changes in the myocardium that are associated with diabetes, characterized by dilatation and hypertrophy of the left ventricle, with the concomitant appearance of diastolic and/or systolic dysfunction, and its presence is in the absence of ischemic heart disease, hypertension, valvular heart diseases and other cardiac pathologies [10–14]. Among multiple mechanisms lead to variant diabetic complications, increased circulating concentrations of lipids and altered tissue metabolism of lipids are consistent features and contribute importantly to cardiovascular complications [6].

The heart balances uptake, metabolism and oxidation of fatty acids to maintain ATP production, membrane biosynthesis and lipid signaling [15]. Although the healthy heart utilizes fatty acids as a source of energy preferentially, T2D causes a further stimulation in fatty acid uptake and oxidation through an elevation in circulating levels of fatty acid and an up-regulation of both fatty acid translocase (FAT/CD36) and fatty acid binding protein, proteins involved in fatty acid uptake by the heart [16, 17]. Additionally, an excessive utilization of fatty acid can cause a rapidly mobilization of triglycerides stores, and limit the degree of glucose oxidation, which may lead to direct damage of myocardium [18].

Generally, lipids are divided into eight classes based on their chemically functional backbones and biochemical principles, including fatty acyls (e.g. fat acids and conjugates, octadecanoids, eicosanoids, docosanoides, and conjugates), glycerolipids (e.g. monoradylglycerols, diradylglycerols and triradyglycerols), glycerophospholipids (e.g. glycerophosphocholines and glycerophosphoglycerols), sphingolipids (e.g. sphingoid bases, ceramides and phosphosphingolipids), sterol lipids (e.g. sterols), prenol lipids (e.g. isoprenoids), saccharolipids (e.g. acrylaminosugars) and polyketides (e.g. linear polyketides) [19, 20]. The fatty acid composition of membrane play an important role in cardiac function [21, 22]. The decrease in affinity of alpha 1-adrenoceptors and the down-regulation of beta-adrenoceptors is accompanied by alteration in percentage fatty acid compositions of phosphatidylethanolamine (PE) and phosphatidylcholine (PC) in cardiac muscle [23].

Previous studies have highlighted the deleterious effect of disorders of lipid metabolism on diabetic heart. The observation of lipids, especially lipidomics profiling, provides an insight into understanding the biomarkers of diabetes and its complications. The aim of our research was to detect changes of myocardial lipidomics profiling in a rat model of diabetic cardiomyopathy using UPLC/Q-TOF/MS analysis, and identify potential biomarkers for further research and clinical diagnosis and therapy in the future.

## Methods

### Chemicals and reagents

Streptozotocin was purchased from Sigma-Aldrich (St Louis, MO, USA), dissolved in citrate buffer (0.1 M, pH = 4.5), and given as a single intraperitoneal (i.p.) injection at a dosage of 30 mg/kg. Liquid chromatography tandem mass spectrometry (LC–MS)-grade acetonitrile, chloroform, and methanol were purchased from Thermo Fisher Scientific (Pittsburgh, PA, USA). Distilled water was acquired from Wahaha purified water company (Hangzhou, China).

### Animal care

All applicable institutional guidelines for the care and use of animals were followed. All procedures performed in studies involving animals were in accordance with the ethical standards of the Institutional Animal Care and Use Committee of Beijing University of Chinese Medicine. Male Sprague–Dawley rats [Grade II, certificate No. SCXK (jing) 2012–0001], weighing 190–200 g, were purchased from Vital River Lab Animal Co. Ltd. (Beijing, China). Animals were adapted for 7 days to an air-conditioned room at a temperature of 23 ± 2 °C, a relative humidity of 55 ± 10%, and a 12-h light–dark cycle (07:00–19:00). They were allowed free access to food and water.

### Diabetic rat model

Fifteen rats were randomly assigned to normal control group and given standard diet, and another 15 rats were fed with high-sucrose/fat diet (HSFD) containing 20% sucrose (w/w), 10% lard (w/w), 2.5% cholesterol (w/w), and 1% bile salt (w/w) in standard feed, which was provided by Ke’ao Cooperation Co. Ltd. (Beijing, China). Rats were fed with HSFD for 6 weeks and then intraperitoneally injected with STZ 30 mg/kg to induce high blood glucose. The rats with fasting blood glucose (FBG) ≥11.1 mmol/L were confirmed as diabetes mellitus 72 h after STZ treatment.

### Measurement of cardiac function

After 22 weeks of injection with STZ (week 22), rats were anesthetized with chloral hydrate at a dosage of 350 mg/kg following a 12-h fast. A catheter (20 G Vasocan Braünle, Penang, Malaysia) was positioned in the left ventricle via the right carotid artery for measurement of left ventricular systolic pressure, left ventricular end diastolic pressure, the maximal uprising velocity of left ventricular pressure (d*p*/d*t*_max_), and the maximal decreasing velocity of left ventricular pressure (d*p*/d*t*_min_). Data were collected using MP150 systems (BIOPAC Systems, Inc. CA, USA).

### Measurement of cardiac index

Following each cardiac function assessment, rat hearts were excised and weighed, and the cardiac index was estimated as follows: cardiac index (%) = whole heart weight/body weight × 100%.

### Measurement of measurement of blood glucose and lipids

Blood samples were obtained from the right carotid artery of rats, and each sample was divided between tubes containing anticoagulant (sodium heparin) and tubes without anticoagulant. Plasma and serum samples were prepared by centrifuging whole blood for 10 min at 2000 g. Fasting glucose, glycosylated serum protein, total cholesterol, triglyceride, and high-density lipoprotein levels in blood samples were determined using an ultraviolet spectrophotometric method according to the manufacturer’s protocol.

### UPLC/Q-TOF/MS analysis

An ultra-high-performance liquid chromatography (UPLC) coupled to quadruple time-of flight (QTOF) mass spectrometer (Waters MS Technologies, Manchester, UK) was used for myocardial tissue sample analysis. Samples were thawed at room temperature, and 100 mg subsamples (100 mg) were accurately weighed and homogenized using a Speed Mill Plus (ANALYTIKJENA, Jena, Germany), and then were extracted using 1000 µL of a chloroform/methanol (3:1, v/v) solution on ice and sonicated for 30 min. After centrifugation (4 °C, 5 min, 13,800 g), 600 µL of supernatant was removed and dried in a Savant Vacuum Concentrator (Thermo Fisher, Pittsburgh, USA). The obtained powder was dissolved in 300 µL isopropyl alcohol/acetonitrile (1:1) for LC-QTOF-MS analysis.

Three microliter of each sample were injected into a Waters Acquity UPLC CSH C_18_ column (2.1 × 50 mm, 1.7 µm) at 55 °C, with the flow rate set at 0.3 mL/min. The water/acetonitrile (3:2, v/v, A)-acetonitrile/isopropanol (9:1, v/v, B) was used as the mobile phase in a gradient elution mode. The elution gradient was 60 ~ 57 ~ 50 ~ 46 ~ 30 ~ 1 ~ 60 ~ 60% (mobile phase A) and 40 ~ 43 ~ 50 ~ 54 ~ 70 ~ 99 ~ 40 ~ 40% (mobile phase B) during 0 ~ 2 ~ 2.1 ~ 12 ~ 12.1 ~ 18 ~ 18.1 ~ 20 min period.

The eluate was analyzed by Waters xevo G2 Q-TOF mass spectrometer in positive mode with a scanning range at m/z 50–1200, applying a capillary voltage of 3.2 kV, and cone voltage of 30 kV. The desolvation gas temperature was set at 400 °C.

### Statistical analysis

All of the MS data were analyzed primitively using the Waters Markerlynx™ XS Application Manager provided with the MassLynx software. Markerlynx Application Manager was employed for data processing including the following steps: detecting chromatographic peak, maximizing ions locating maximizing ions, assembling ion data into a matrix. Markerlynx preprocessed data was imported to EZinfo 2.0 software (Waters Corporation, Manchester, UK) for principal component analysis (PCA) and orthogonal projection to latent structures discriminant analysis (OPLS-DA). The potential markers were extracted from S-Plots constructed following OPLS analysis based on their contribution to the variation and correlation. And HMDB (http://www.hmdb.ca/), MetaboAnalyst (http://www.metaboanalyst.ca/), and LIPID MAPS (http://www.Lipidmaps.org/tools/index.html) were used to identify the selected potential biomarkers.

Additional statistical analysis was conducted in SPSS version 17.0. Data are presented as mean ± standard error of the mean (SEM). Welch’s *t* test was used to test whether these parameters differed between DCM and controls. Correlation studies were done using Pearson Correlation test when appropriate. Values of *P* < 0.05 were considered statistically significant.

## Results

### Multivariate statistical analysis of UPLC/Q-TOF/MS data

The UPLC/Q-TOF/MS system was used to acquire the metabolic profiles of myocardial tissue of the control and DCM ratss in ESI^+^ and ESI^−^ modes. From the original data, 3023 peaks of positive ions and 300 peaks of negative ions were detected and analyzed by Marker Lynx using the single acquisition method. In order to gain an overview of the metabolic profiling of myocardial tissue, PCA and OPLS-DA score plots were used in the subsequent UPLC/Q-TOF/MS data analysis. The PCA scores were the weights of averages of the original data, which could provide a good summary of all variables. OPLS-DA scores were calculated to differentiate between the DCM and control rats. In this study, small molecule metabolites of myocardial tissue in DCM model and control rats were shown on OPLS-DA score plots. Compared with control rats, the metabolic profiles of myocardial tissue in DCM model rats were significantly altered, which might be related to hyperglycemia and hypercholesterolemia status.

**Fig. 1:**
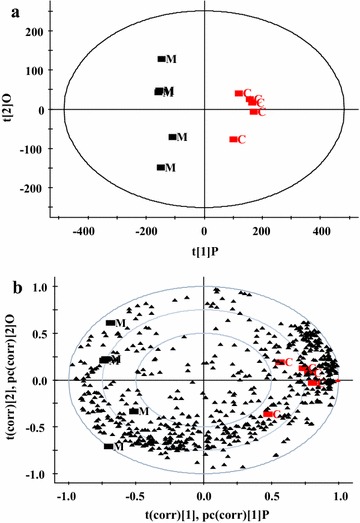
OPLS-DA score plot and loading plot. Scatter plots of diabetic cardiomyopathy (DCM) model and control rats (n = 5 per group) were acquired by ultra-high-performance liquid chromatography coupled with quadruple time-of-flight mass spectrometry (UPLC/Q-TOF/MS) in ESI^+^ mode: **a** OPLS-DA score plot. **b** OPLS-DA loading plot in ESI^+^ mode and variables labeled with retention time. (*Black square*): DCM model rat, M; (*black square*): *c* Control rat, *OPLS-DA* orthogonal projection to latent structures discriminant analysis, *ESI* electrospray mode electrospray ionization

In addition, an OPLS-DA loading plot was generated. The loading plot displays both the score values of our observations and weights of the variables, which could be used to find potential biomarkers. The results manifested quantitative differences in metabolites between DCM and control rats (Fig. 1).

### Score plot

A score plot (S-Plot) is a tool for visualizing both the covariance and correlation between the endogenous metabolites and the modeled class designation. Thus, an S-Plot could be used to identify biochemically atypical metabolites with statistical significance, based on contributions both of the model and their reliabilities.

**Fig. 2:**
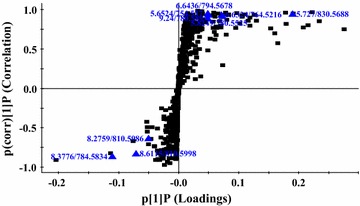
Score plot (S-Plot) of OPLS-DA pattern. Variables were considered to be significant to the model when the VIP score was above 1.0; *OPLS-DA* orthogonal projection to latent structures discriminant analysis

The contribution of the variables to the model can be expressed using a variable importance in projection (VIP) score. Typically, a variable is considered to be significant to the model when the VIP score is above 1.0. In this study, in order to highlight significant variables, only those with VIP scores above 1.0 and errors below 1.0 were adopted. The S-Plot and VIP scores of the OPLS/DA data are given in Fig. 2. A typical metabolites related to the group separation were selected as potential biomarkers from the S-Plots.

### Potential biomarkers

Metabolites that showed differences between groups (*t* test, *P* < 0.05) were analyzed by MetaboAnalyst 3.0 software (http://www.metaboanalyst.ca/). Structures of the compounds were then identified by analyzing mass spectra using HMDB (http://www.hmdb.ca/) and LIPID MAPS (http://www.lipidmaps.org/). Nine potential biomarkers were identified. Compared with control rats, the levels of PC (22:6/18:2), PC (22:6/18:1), PC (20:4/16:1), PC (16:1/18:3), PE (20:4/18:2), and PE (20:4/16:0) of DCM model rats were significantly down-regulated, and the levels of PC (20:2/18:2), PC (18:0/16:0), and PC (20:4/18:0) or PC (20:3/18:1) were significantly up-regulated (Table 1), most of which were metabolic intermediates of glycerophospholipid metabolism.

**Table 1:** Potential biomarkers of diabetic cardiomyopathy in rats

No.	t_R_ (min)	Mass-to-charge (m/z) ratios	VIP values	P value	DCM model versus control	Chemical names	Structure	Fold values
Positive mode								
1	5.7270	830.5688	4.75926	0.000298	↓	PC (22:6/18:2)	C48H80NO8P	2.3550
2	6.2368	832.5842	1.82151	0.000235	↓	PC (22:6/18:1)	C48H82NO8P	2.0013
3	6.6324	764.5216	1.83260	8.78E−05	↓	PE (20:4/18:2)	C43H74NO8P	2.1611
4	8.2759	810.5986	1.21197	0.041959	↑	PC (20:2/18:2)	C46H84NO8P	2.3574
5	8.3776	784.5834	2.92017	0.000513	↑	PC (18:0/16:0)	C42H84NO8P	4.3254
6	8.6115	810.5998	1.89759	1.46E−05	↑	PC (20:4/18:0) or PC (20:3/18:1)	C46H84NO8P	6.7733
7	5.8249	780.5525	1.86694	0.001519	↓	PC (20:4/16:1)	C44H78NO8P	3.3939
8	5.6524	754.5370	1.27221	0.000592	↓	PC (16:1/18:3)	C42H76NO8P	4.4684
9	9.2400	780.5890	1.23109	0.001364	↓	PE (20:4/16:0)	C41H74NO8P	3.3926

PC, phosphatidylcholine; PE, phosphatidylethanolamine; t_R_, retention time; VIP, variable importance in projection; DCM, diabetic cardiomyopathy

### Blood glucose and lipids

When compared with control rats, fasting blood glucose, glycosylated serum protein, total cholesterol, and triglyceride levels of DCM rats were significant increased by 368, 34, 230, and 92%, respectively at week 22 (*P* < 0.001, *P* < 0.05, *P* < 0.01, *P* < 0.01). High-density lipoprotein level was remarkably decreased by 46% in DCM rats (*P* < 0.05) (Table 2).

**Table 2:** Blood glucose and lipids

Group	Fast blood glucose (mmol/L)	Glycosylated serum protein (mmol/L)	Cholesterol (mmol/L)	Triglycerides (mmol/L)	High-density lipoprotein (mmol/L)
Control	5.081 ± 0.492	3.653 ± 0.970	1.463 ± 0.159	0.472 ± 0.101	1.011 ± 0.369
DCM model	23.813 ± 2.880***	4.895 ± 0.692*	4.836 ± 1.174**	0.908 ± 0.290**	0.538 ± 0.167*

Values given are mean ± SEM, with n = 12

DCM, diabetic cardiomyopathy

* *P* < 0.05, ** *P* < 0.01, *** *P* < 0.001 versus control group

### Cardiac function and mass

Left ventricular systolic pressure and d*p*/d*t*_max_ in DCM model rats were significant decreased (by 21 and 38%, *P* < 0.05, respectively), when compared with the control rats. Left ventricular end diastolic pressure and d*p*/d*t*_min_ in DCM model group significantly increased by 35 and 44% (*P* < 0.05), when compared with the control rats. Compared with the control rats, the water consumption and cardiac index of DCM model rats was increased by 524 and 25% (*P* < 0.001, *P* < 0.01), while the body weight remarkably decreased by 26% (*P* < 0.01), indicating cardiac hypertrophy in the DCM model group (Table 3).

**Table 3:** Cardiac function and mass

Group	LVSP (mmHg)	d*p*/d*t*_max_ (mmHg/s)	LVEDP (mmHg)	d*p*/d*t*_min_ (mmHg/s)	Whole heart weight (g)	Body weight (g)	Whole heart weight/body weight (%)	Water consumption (mL/24 h)
Control	112 ± 17	5822 ± 1740	−0.08 ± 1.30	−7680 ± 1866	1.389 ± 0.185	574 ± 35	0.24 ± 0.026	33 ± 9
DCM model	88 ± 14*	3637 ± 733*	2.78 ± 2.32*	−4269 ± 1076*	1.269 ± 0.105	426 ± 56**	0.30 ± 0.020**	206 ± 50***

Values given are mean ± SEM, with n = 12

DCM, diabetic cardiomyopathy; LVSP, left ventricular systolic pressure; d*p*/d*t*_max_, the maximal uprising velocity of left ventricular pressure; LVEDP, left ventricular end diastolic pressure; d*p*/d*t*_min_, the maximal decreasing velocity of left ventricular pressure

* *P* < 0.05, ** *P* < 0.01, *** *P* < 0.001 versus control group

### Relationship of cardiac lipid-related biomarkers to cardiac function and mass parameters

**Fig. 3:**
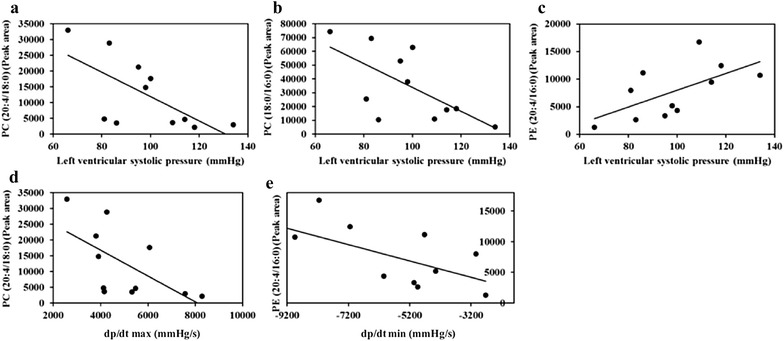
Relationship of biomarkers to cardiac function parameters in vivo. **a** Left ventricular systolic pressure and PC (20:4/18:0). Pearson correlation analysis shows a significant negative correlation of left ventricular systolic pressure versus peak area of PC (20:4/18:0) (Pearson r = −0.663; *P* = 0.026, *n* = 11).* Line* represents linear regression of data (y = −385.9x + 50504; r^2^ = 0.4402). **b** Left ventricular systolic pressure and PC (18:0/16:0). Pearson correlation analysis shows a significant negative correlation of left ventricular systolic pressure versus peak area of PC (18:0/16:0) (Pearson r = −0.655; *P* = 0.029, *n* = 11). *Line* represents linear regression of data (y = −864.51x + 120262; r^2^ = 0.4291). **c** Left ventricular systolic pressure and PE (20:4/16:0). Pearson correlation analysis shows a significant positive correlation of left ventricular systolic pressure versus peak area of PE (20:4/16:0) (Pearson r = 0.612; *P* = 0.045, *n* = 11). *Line* represents linear regression of data (y = 151.95x − 7199.9; r^2^ = 0.3749). **d** d*p*/d*t*_max_ and PC (20:4/18:0). Pearson correlation analysis shows a significant negative correlation of d*p*/d*t*_max_ versus peak area of PE (20:4/18:0) (Pearson r = −0.623; *P* = 0.041, *n* = 11). *Line* represents linear regression of data (y = −4.1053x + 33206; r^2^ = 0.3883).** e** d*p*/d*t*_min_ and PC (20:4/16:0). Pearson correlation analysis shows a significant negative correlation of d*p*/d*t*_min_ versus peak area of PC (20:4/16:0) (Pearson r = −0.63; *P* = 0.038, *n* = 11). *Line* represents linear regression of data (y = −1.3289x − 26.661; r^2^ = 0.3965)

To explore the relationship of potential cardiac lipid-related biomarkers to cardiac function and mass parameters, a correlational analysis was performed for all rats, and the results indicated a remarkable correlation of cardiac lipid-related biomarkers with changes of cardiac function and mass in DCM rats. Among 9 potential cardiac lipid-related biomarkers showed in Table 1, 5 of which have significant correlation with cardiac function parameters. In the heart, peak area of PC (20:4/18:0) and PC (18:0/16:0) negatively correlated to left ventricular systolic pressure, PC (20:4/18:0) negatively correlated to d*p*/d*t*_max_, PC (20:4/16:0) negatively correlated to d*p*/d*t*_min_ (*P* < 0.05). Meanwhile, PE (20:4/16:0) positively correlated to left ventricular systolic pressure (*P* < 0.05) (Fig. 3).

**Fig. 4:**
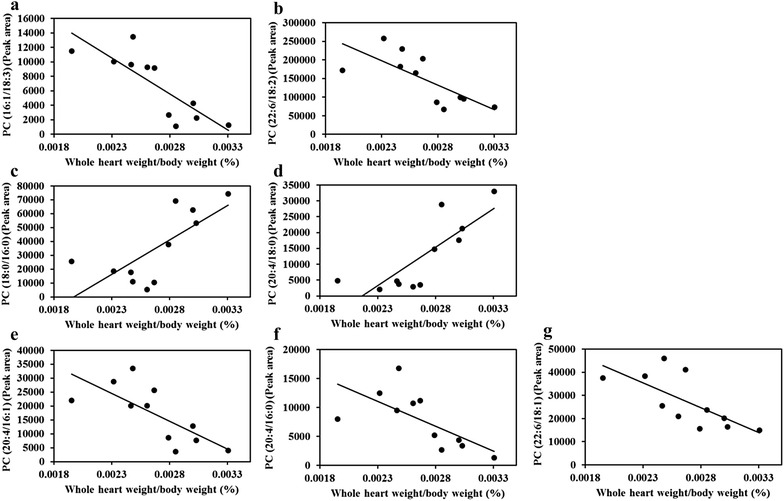
Relationship of biomarkers to the ratio of whole heart weight/body weight in vivo. **a** Whole heart weight/body weight and PC (16:1/18:3). Pearson correlation analysis shows a significant negative correlation of the ratio of whole heart weight/body weight versus peak area of PC (16:1/18:3) (Pearson r = −0.828; *P* = 0.002, *n* = 11). *Line* represents linear regression of data (y = −10^−7^x + 33438; r^2^ = 0.6856). **b** Whole heart weight/body weight and PC (22:6/18:2). Pearson correlation analysis shows a significant negative correlation of the ratio of whole heart weight/body weight versus peak area of PC (22:6/18:2) (Pearson r = −0.733; *P* = 0.01, *n* = 11). *Line* represents linear regression of data (y = −10^−8^x + 498642; r^2^ = 0.5377). **c** Whole heart weight/body weight and PC (18:0/16:0). Pearson correlation analysis shows a significant positive correlation of the ratio of whole heart weight/body weight versus peak area of PC (18:0/16:0) (Pearson r = 0.727; *P* = 0.011, *n* = 11). *Line* represents linear regression of data (y = 5 × 10^−7^x − 97683; r^2^ = 0.5281). **d** Whole heart weight/body weight and PC (20:4/18:0). Pearson correlation analysis shows a significant positive correlation of the ratio of whole heart weight/body weight versus peak area of PC (20:4/18:0) (Pearson r = 0.808; *P* = 0.003, *n* = 11). *Line* represents linear regression of data (y = 2 × 10^−7^x − 52535; r^2^ = 0.6521). **e** Whole heart weight/body weight and PC (20:4/16:1). Pearson correlation analysis shows a significant negative correlation of the ratio of whole heart weight/body weight versus peak area of PC (20:4/16:1) (Pearson r = −0.731; *P* = 0.011, *n* = 11). *Line* represents linear regression of data (y = −2 × −10^−7^x + 70585; r^2^ = 0.5345). **f** Whole heart weight/body weight and PC (20:4/16:0). Pearson correlation analysis shows a significant negative correlation of the ratio of whole heart weight/body weight versus peak area of PC (20:4/16:0) (Pearson r = −0.671; *P* = 0.024, *n* = 11). *Line* represents linear regression of data (y = −9 × −10^−6^x + 30834; r^2^ = 0.4507). **g** Whole heart weight/body weight and PC (22:6/18:1). Pearson correlation analysis shows a significant negative correlation of the ratio of whole heart weight/body weight versus peak area of PC (22:6/18:1) (Pearson r = −0.711; *P* = 0.014, *n* = 11). *Line* represents linear regression of data (y = −2 × −10^−7^x + 84752; r^2^ = 0.5056)

Among 9 potential cardiac lipid-related biomarkers showed in Table 1, 7 of which have significant correlation with the ratio of whole heart weight/body weight. In the heart, peak area of PC (16:1/18:3), PC (22:6/18:2), PC (20:4/16:1), PC (20:4/16:0) and PC (22:6/18:1) negatively correlated to the ratio of whole heart weight/body weight (*P* < 0.01, *P* < 0.05, *P* < 0.05, *P* < 0.05, *P* < 0.05), and peak area of PC (18:0/16:0) and PC (20:4/18:0) positively correlated to the ratio of whole heart weight/body weight (*P* < 0.05, *P* < 0.01) (Fig. 4).

## Discussion

The ectopic disposition of lipids may be a cause of heart failure when suffered from obesity and type 2 diabetes mellitus. The previous studies have clearly shown a correlation between the accumulation of triglycerides and heart dysfunction [24]. In this study, we have analyzed the myocardial lipodomics profiling of the diabetic heart, and the results manifested that phospholipids (e.g. phosphatidylcholines and phosphatidylethanolamines) play an important role in the development of diabetic cardiomyopathy.

Phospholipids are typically divided into two categories in the body: glycerophospholipids (e.g. phosphatidylcholines, phosphatidylethanolamines, phosphatidylserines and phosphatidylinositols) and sphingomyelin. Glycerophospholipids are synthesized de novo in the fed state by the esterification of glycerol 3-phosphate originating from glycerol or dihydroxyacetone-phosphate. This process is located in the endoplasmic reticulum and to a lesser extent in the mitochondria [25]. Phosphatidylcholines (PCs) are generally the most abundant phospholipid in a membrane, which are composed of two fatty acids covalently linked to a glycerol moiety by ester bonds in the sn-1 and sn-2 positions [26]. Phospholipids are involved in many cell processes including membrane trafficking, signal transduction, and autophagy-mediated protein degradation. Membrane phospholipids are also the reservoir of lipid mediators and signaling molecules such as arachidonic acid, prostaglandins, inositol triphosphate, endocannabinoids, and diacylglycerol [25].

The structure and nature of glycerophospholipids and sphingomyelin are similar. Sphingolipids are ubiquitous eukaryotic membrane lipids, present particularly in the myelin sheath of neuronal cells. They include sphingomyelins and glycosphingolipids [27], including the cerebrosides, sulfatides, globosides, and gangliosides. Long-chain fatty acid and very long-chain fatty acid, with different chain structures and saturations are needed for the biosynthesis of phospholipids, plasmalogens, and glycosphingolipids, as well as the remodeling of their acyl chains [28, 29].

In male rats, total phospholipids in the heart predominantly contain 18:0, 18:2n−6, and 20:4n−6, while triacylglycerol [30] was enriched in 16:0, 18:1n−9, and 18:2n−6 [31]. Sex is an important factor in determining the effects of dietary fat on the fatty acids composition of the heart. The ranking between lipid classes in number of fatty acids altered by the interaction of fat intake and sex was PC > PE > triacylglycerol [32]. The oleate and vaccenate turnover were the highest in cardiolipin, whereas palmitate and stearate turnover were low in rats fed a high-fat diet [33].

Obesity is associated with an elevated risk of chronic disease, including diabetes and cardiovascular disease [34–36]. Diastolic dysfunction is often the earliest functional cardiac abnormality associated with obesity [37]. With obesity and diabetes, hearts are likely to have metabolic imbalances and lipid accumulation. Western-style diets enriched with fats such as palmitic acid (C16:0) and oleic acid (C18:1) have been linked to an increased risk of type 2 diabetes [35]. In cardiomyocytes, plasma lipids can be taken up as fatty acids to undergo mitochondrial β-oxidation for energy supply. An overload of lipid utilization causes increased oxidation and reactive oxygen species, mitochondrial damage, and apoptosis. Fatty acids may also accumulate as triacylglycerol and phospholipids in lipid droplets, or deviate to ceramides [38]. After 4 weeks of feeding a lipid-supplemented diet (20% w/w) containing sunflower oil/lard (1:1), adult rats showed decreased cardiolipin content and increased lysophosphatidylcholine and PC contents compared with control rats. The polyunsaturated/saturated fatty acids ratio was also lower in the lipid-supplemented rats compared with control rats [39]. High levels of free fatty acids may increase myocardial ischemic damage. However, in the study of the effects of fatty acids and phospholipids in lipid emulsions on stunned myocardium, Van de Velde et al. found that administering Intralipid^®^ during reperfusion improved recovery in contractile function and increased the high-energy phosphate content. Both linoleic acid and PC significantly improved myocardial function in stunned myocardium [40]. In diabetic hearts, ectopic lipid accumulates and contributes to diastolic dysfunction. A distinct pattern of myocardial lipid remodeling, accompanied by oxidative stress, is associated with the onset of diastolic dysfunction in obese insulin-resistant *db*/*db* mice. Five of the nine individual lipid subspecies (C14:1, C16:1, C16:0, C18:1, and C20:4) were elevated in 12-week-old *db*/*db* hearts and six of nine (C14:1, C16:1, C16:0, C18:1, C20:4, and C22:6) were increased in 15-week-old *db*/*db* hearts [41].

## Conclusions

UPLC/Q-TOF/MS analysis data suggested changes of some potential lipid biomarkers in the development of cardiac dysfunction and hypertrophy of diabetic cardiomyopathy, including down-regulation of PC (22:6/18:2), PC (22:6/18:1), PC (20:4/16:1), PC (16:1/18:3), PE (20:4/18:2), and PE (20:4/16:0), and up-regulation of PC (20:2/18:2), PC (18:0/16:0), and PC (20:4/18:0). Knowledge of these differences in the metabolite profile may be useful to understanding the pathogenesis of DCM, and these results may also serve as potential important targets for clinical diagnosis and therapeutic intervention of DCM in the future.

## Abbreviations

DCM: diabetic cardiomyopathy
HSFD: high-sucrose/fat diet
LVEDP: left ventricular end diastolic pressure
LVSP: left ventricular systolic pressure
d*p*/d*t*_min_: the maximal uprising velocity of left ventricular pressure
OPLS-DA: orthogonal projection to latent structures discriminant analysis
PCA: principal component analysis
PC: phosphatidylcholine
PE: phosphatidylethanolamine
d*p*/d*t*_max_: the maximal decreasing velocity of left ventricular pressure
STZ: streptozotocin
UPLC/Q-TOF/MS: ultra-high-performance liquid chromatography (UPLC) coupled to quadruple time-of flight (QTOF) mass spectrometer
